# Dissecting the expression landscape of cytochromes P450 in hepatocellular carcinoma: towards novel molecular biomarkers

**DOI:** 10.18632/genesandcancer.190

**Published:** 2019-05

**Authors:** Camille Martenon Brodeur, Philippe Thibault, Mathieu Durand, Jean-Pierre Perreault, Martin Bisaillon

**Affiliations:** ^1^ Département de biochimie, Faculté de médecine et des sciences de la santé, Université de Sherbrooke, Sherbrooke, Québec, Canada; ^2^ Laboratoire de Génomique Fonctionnelle, Université de Sherbrooke, Sherbrooke, Quebec, Canada

**Keywords:** hepatocellular carcinoma, cytochromes, gene expression, biomarker

## Abstract

Hepatocellular carcinoma (HCC) is the second leading cause of cancer-related deaths around the world. Recent advances in genomic technologies have allowed the identification of various molecular signatures in HCC tissues. For instance, differential gene expression levels of various cytochrome P450 genes (CYP450) have been reported in studies performed on limited numbers of HCC tissue samples, or focused on a small subset on CYP450s. In the present study, we monitored the expression landscape of all the members of the CYP450 family (57 genes) in more than 200 HCC tissues using RNA-Seq data from The Cancer Genome Atlas. Using stringent statistical filters and data from paired tissues, we identified significantly dysregulated CYP450 genes in HCC. Moreover, the expression level of selected CYP450s was validated by qPCR on cDNA samples from an independent cohort. Threshold values (sensitivity and specificity) based on dysregulated gene expression were also determined to allow for confident identification of HCC tissues. Finally, a global look at expression levels of the 57 members of the CYP450 family across ten different cancer types revealed specific expression signatures. Overall, this study provides useful information on the transcriptomic landscape of CYP450 genes in HCC and on new potential HCC biomarkers.

## INTRODUCTION

Hepatocellular carcinoma (HCC) is the cancer with the second highest mortality rate worldwide [1]. It is generally associated with risk factors such alcohol consumption and aflatoxin B1 exposition [2]. HCC occurrence is still rising even in developed countries where it is linked with obesity, diabetes, and hepatitis B and C virus (HBV and HCV) infection [3]. The poor survival rate of HCC patients (1 and 5-year survival rate of 44% and 17%, respectively [4]) is partly due to limited treatments options and their unsatisfactory efficacy. Liver transplantation or chirurgical resection of the tumor are the only curative treatments [5]. However, since HCC diagnosis is generally tardive, more than 80% of patients are not eligible and chemoembolization or drugs have to be used. Nonetheless, these treatments have limited efficacy at advanced tumor stages [6]. Hence, early diagnosis of patients for hepatocellular carcinoma is crucial.

HCC screening is generally made by imagery techniques, such as ultrasound or computed tomography (limited to tumor bigger than 1 cm), or by assessing the alpha-fetoprotein (AFP) serum levels [7, 8]. AFP is a glycoprotein produced by fetal yolk sac and liver, and its concentration decreases rapidly after birth. Some conditions, like pregnancy or cancer, can generate high AFP levels in serum [9]. AFP level can give information about HCC since it is positively correlated with HBV infection, tumor size, low cellular differentiation, and reaches the highest level in the case of metastatic tumor [10–12]. However, its low expression in early stage cancers makes it a poor biomarker for large-scale HCC screening, especially since 30% of patients with HCC continuously have normal AFP levels [13]. Other pathologies such as cirrhosis and stomach cancer also have elevated AFP levels which contribute to the relative non-specificity for HCC detection [14]. This leads to sensitivity levels which vary from 55% to 61%, and specificity levels of 78% to 91% when a 20 ng/mL level cut-off is used for HCC screening [15].

The low sensibility and high false-positive rates of AFP as marker therefore justify the identification of better biomarkers for HCC screening. The development of new technologies, notably in genomics, allows characterization of molecular events involved in carcinogenesis, including mRNA expression levels in tissue samples. The Cancer Genome Atlas (TCGA) research network recently overviewed HCC and normal liver tissues data obtained from multiple genomic platforms. They identified important characteristics of HCC such as significantly mutated genes (e.g. CTNNB1A, TP53, TERT promoter), different promoter methylation profiles (hypermethylation of CDKN2A which causes gene silencing), and key pathways affected in HCC (WNT, SHH, RTK/KRAS, chromatin remodeling and metabolic programming) [3]. In the current large-scale study, RNA sequencing data from the TCGA were used to identify differentially expressed genes in more than 200 HCC tissues. Our study primarily focuses on members of the cytochrome P450 family in order to find potential biomarkers.

## RESULTS

### Modification of the gene expression landscape in hepatocellular carcinoma

**Figure 1:**
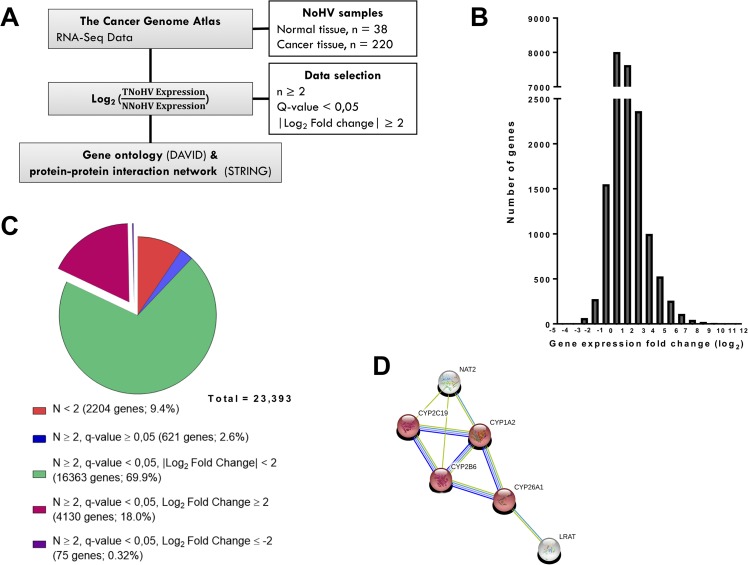
Transcriptomic study of hepatocellular carcinoma **A.** Overview of the strategy used to identify the changes in cellular transcriptome of hepatocellular carcinoma (HCC) tumors and the potential biomarkers genes, based on the RNA-Seq data of 220 HCC and 38 normal liver tissues, both without viral infection, from The Cancer Genome Atlas (TCGA). N: Normal, T: Tumor, NoHV: no hepatitis virus infection, HBV: hepatitis B virus-infected tissue, HCV: hepatitis C virus-infected tissue, HBCV: hepatitis B and C virus-infected tissue. **B.** Distribution of HCC gene expression levels as compared to normal liver tissues. The variations in gene expression are presented in a logarithmic scale (log2). **C.** Statistical analysis of gene expression fold change to identified significantly dysregulated genes in HCC. 4,130 overexpressed (magenta) and 75 repressed (purple) genes were selected for further analysis. **D.** Gene node with monooxygenase activity (protein-protein interaction enrichment p-value: 1.93e-14; false discovery rate: 0.0254) found in the protein-protein interaction network of 75 genes for which the expression was repressed in HCC. The complete network is presented in figure S1B. The network was determined by uploading the gene list into STRING [43].

During carcinogenesis, cancer cells acquire multiple types of alterations, such as mutations, that modify the transcription of target genes. The resulting gene expression differences can then be used to discriminate between normal and cancer cells. In this study, the gene expression profile of HCC tissues was compared to the transcriptome of normal liver tissues based on RNA sequencing data from The Cancer Genome Atlas (TCGA) in order to identify new potential biomarkers. The overview of the steps used towards the identification of such differences in gene expression is presented in Figure 1A. To focus on transcriptomic changes resulting from carcinogenesis and not from viral infection, analyses were made on non-infected normal tissues (normal, no hepatitis virus; NNoHV) and non-infected tumor tissues (tumor, no hepatitis virus; TNoHV). Average gene expression, in transcript-per-millions (TPM), of 220 TNoHV tissues was then compared to the average expression of 38 NNoHV tissues to establish differential gene expression levels (fold-change presented in base 2 logarithm). The distribution of gene expression levels (Figure 1B) revealed that the majority of genes have similar expression levels in both conditions, although some have a variation of expression that reaches over 3,000-fold in tumors. The gene list, containing 23,393 genes detected either in HCC or normal tissues, was then filtered to select differentially expressed genes. Genes detected in at least two samples in both conditions were kept and, to correct for multiple statistical hypothesis testing, q-value were calculated, and values inferior to 0.05 were considered significant. Finally, a fold-change of at least four was chosen as a cut-off to be suitable to distinguish HCC from normal tissues. This allowed the identification of 4,130 overexpressed and 75 repressed genes in HCC (Figure 1C). Gene ontology analysis of the overexpressed genes, using DAVID, indicates enrichment in biological processes generally associated with carcinogenesis such as cell-cell signaling, cell adhesion, cell proliferation and cell cycle processes. Among repressed genes, gene ontology analysis showed immune and defense response as the most enriched biological processes (Supplementary Figure 1A), as observed in a previous study based on the Gene Expression Omnibus database using a smaller number of samples (38 HCC samples and 19 normal samples) [16]. In the case of repressed genes, a protein-protein interaction network was generated using STRING. This analysis revealed the presence of a network of six proteins encompassing four members of the cytochrome P450 (CYP450) family (Figure 1D). Interestingly, vitamin A, diterpenoid, and retinoid metabolic processes, which are all associated with CYP450, were also observed in the gene ontology analysis performed on repressed genes (Supplementary Figure 1A).

### Cytochromes P450 expression profile in hepatocellular carcinoma

**Figure 2:**
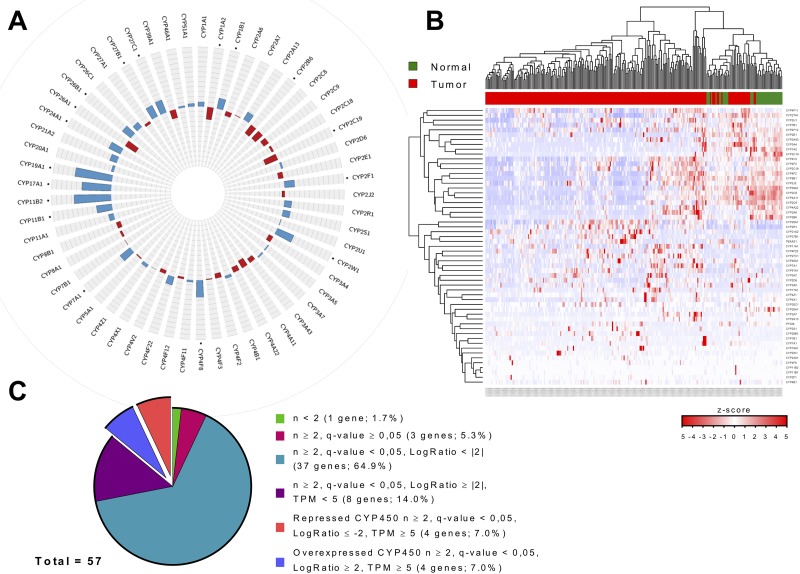
Global profiling of the cytochromes P450 expression landscape in hepatocellular carcinoma **A.** Gene expression variation of the 57 cytochromes P450 in tumor tissues compared to normal tissues (non-infected by hepatitis viruses). Dots indicate fold-change (in log2) of at least −2 and 2. Overexpression values are shown in blue and repression values in red, and the scale is from −12 to 12. **B.** Heatmap representation of cytochromes P450 gene expression for each normal (green) and tumor (red) samples (non-infected). Red indicates high levels and blue indicates low levels of gene expression. **C.** The cytochromes P450 list was filtered to keep genes with significant expression variation in HCC. Using this approach, we selected eight CYP450s; four of them were up-regulated (blue; CYP1B1, CYP7A1, CYP17A1, and CYP19A1), while four were down-regulated (orange; CYP1A2, CYP2B6, CYP2C19, and CYP26A1) in tumor cells compared to normal cells.

Differential gene expression levels of various CYP450s have previously been reported [23–26]. However, these previous studies were generally performed on a limited number of HCC tissue samples or focused on a small subset on CYP450s [23–26]. In humans, 57 CYP450 genes are expressed in multiple tissues such as liver, placenta, brain, kidney, and intestines where they catalyze monooxygenase activity on specific substrates [17, 18]. Some CYP450 proteins participate in the metabolism of xenobiotics in the liver such as environmental chemicals or therapeutic drugs. Other CYP450s have a role in the biotransformation of endogenous compounds like cholesterol, steroid hormones, bile acids, or eicosanoids [17, 19]. Since some members of the CYP450 family showed enrichment among dysregulated genes in HCC, we monitored the expression landscape of all the members of the CYP450 family (57 CYP450 genes). Following statistical analysis, 17 out of 57 CYP450s were found to have fold-changes of at least four in HCC (Figure 2A). It should be noted that expression level variation of CYP450s is relatively constant between HBV-, HCV-, or HBCV-infected tumors compared to non-infected tumors (*r* = 0.76-0.88) (Supplementary Figure 2). A global look at the CYP450 expression levels across normal and HCC tissues shows that sample types (i.e. normal or cancer) have a tendency to cluster together, suggesting that HCC tissues could be identified according to their CYP450 expression levels (Figure 2B).

Statistical filtering was then applied to select significantly dysregulated CYP450s. Again, genes detected in at least two samples in both conditions were kept and, to correct for multiple statistical hypothesis testing, q-value were calculated (values inferior to 0.05 were considered significant). Because some of the RNA transcripts were detected at a low level in every sample, a criterion of a mean expression of at least 2 TPM in one or the other condition (normal or cancer) was added to make sure the transcript would be detectable in screening assays. Our analysis revealed that CYP1B1, CYP7A1, CYP17A1, and CYP19A1 were significantly overexpressed in HCC, while CYP1A2, CYP2B6, CYP2C19, and CYP26A1 were significantly repressed in HCC, compared to their expression in normal liver tissues (Figure 2C).

**Figure 3:**
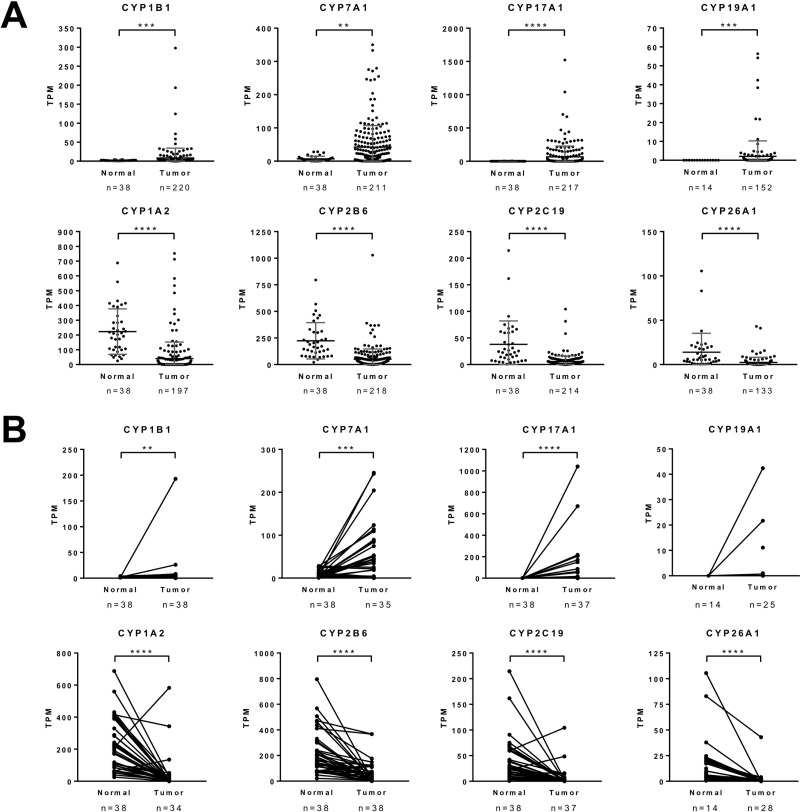
Specific gene expression analysis of the eight cytochromes P450 between hepatocarcinoma and normal tissues **A.** Gene expression, in transcripts per million (TPM), in each tumor and normal tissues samples, of the eight selected CYP450s that were significantly up- or down-regulated. Grey lines indicate mean ± standard deviation in each groups. Mann-Whitney U test. **B.** Gene expression, in TPM, of the eight selected CYP450s in paired tumor and normal tissues samples. Wilcoxon rank sum test. ^*^ = *p* < 0.05; ^**^ = *p* < 0.01; ^***^ = *p* < 0.001; ^****^ = *p* < 0.0001.

Since fold-changes were calculated from average expression levels in normal and tumor tissues, the expression of the eight selected CYP450s in individual samples was investigated. This revealed that the global gene expression distribution was still significantly different between normal and tumor tissues (Figure 3A). Moreover, to confirm that differences in gene expression are not due to interindividual variability, the expression level of each gene was evaluated in paired tissues (normal and tumor) (Figure 3B). Up- and down-regulation of candidate in cancer tissues was still statistically significant different in cancer tissues for the selected CYP450s, except for CYP19A1. This is attributed to the fact that CYP19A1 was not detected in the majority of the paired tissue samples, although two paired samples exhibit a high overexpression in tumors. HCC samples were then separated according to their pathologic stage to evaluate the expression variation of the eight selected CYP450s during carcinogenesis (stages I to IV). The analysis shows that the observed change of expression is relatively constant from stage I to stage IV (Supplementary Figure 3A).

### Candidate cytochromes P450 expression validation

**Figure 4:**
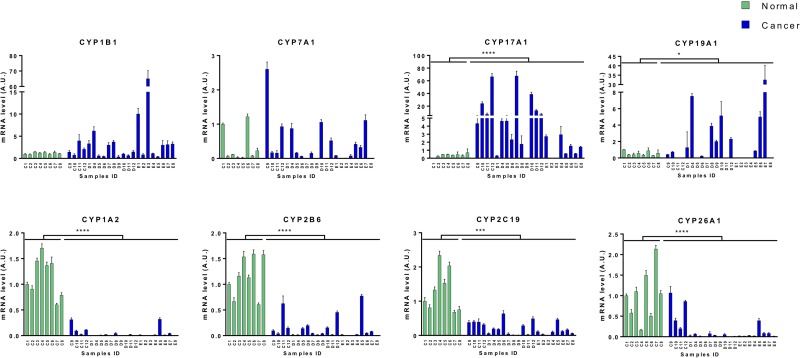
Validation of cytochromes P450 expression variation in HCC Expression of up-regulated and down-regulated CYP450s in HCC and normal tissue cDNA array by qPCR. Mean expression and standard deviation for each sample were obtained from three technical replicates and are plotted in arbitrary units. Significant variation between both groups (8 normal samples (C1 to C8, green) and 22 HCC samples (C9 to E8, blue)) are shown. Analysis of the six paired samples is available in supplementary data (Supplementary Figure 4). Mann-Whitney U test. ^*^ = *p* < 0.05; ^**^ = *p* < 0.01; ^***^ = *p* < 0.001; ^****^ = *p* < 0.0001.

To validate the differential expression of CYP450 previously identified by RNA-Seq from the TCGA dataset, qPCR assays were performed on an independent cohort. Commercially available cDNA plates were generated from 8 normal and 22 HCC tissues from which total mRNAs were extracted and converted into cDNA. Among those samples, there are 6 paired samples which allowed validation of intraindividual expression variation (Supplementary Figure 4). These qPCR results confirmed a similar distribution of gene expression; overexpression of CYP1B1, CYP7A1, CYP17A1, and CYP19A1, and repression of CYP1A2, CYP2B6, CYP2C19, and CYP26A1 was observed in cancer tissues (Figure 4). However, since some tumors have relatively normal gene expression level and there is interindividual variability in samples, the differences in CYP1B1 and CYP7A1 expression levels between normal and cancer tissues were not considered statistically significative.

### Potential diagnosis of candidate CYP450s

**Figure 5:**
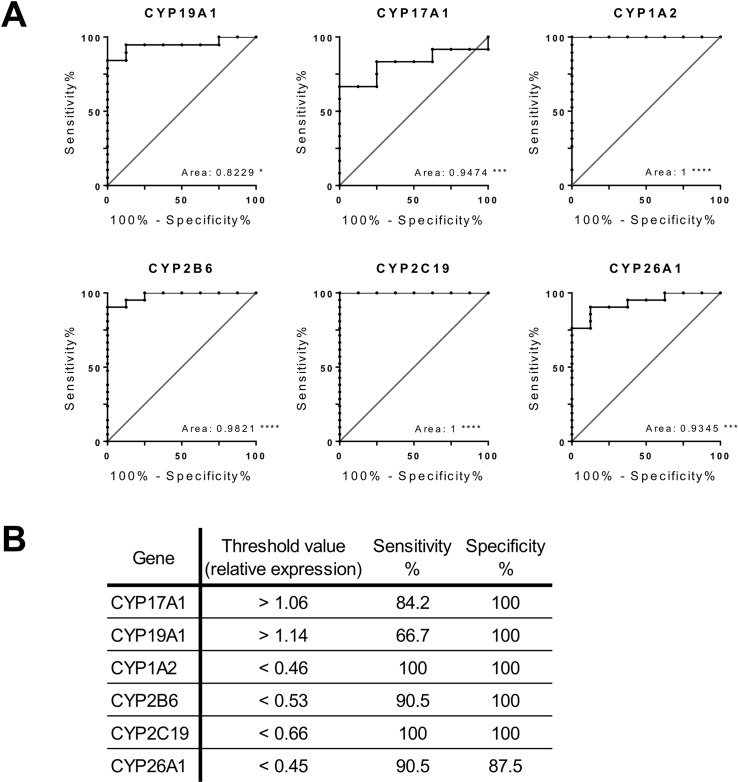
Receiver Operating Characteristic (ROC) curve of candidates cytochromes P450 for HCC identification **A.** ROC curve for relative expression of HCC (*n* = 22) and normal (*n* = 8) cDNA samples for each validated dysregulated genes (CYP17A1, CYP19A1, CYP1A2, CYP2B6, CYP2C19, and CYP26A1). The corresponding area under the curve (AUC) value is indicated. Diagonal lines represent the performance of a random classifier. ^*^ = *p* < 0.05; ^**^ = *p* < 0.01; ^***^ = *p* < 0.001; ^****^ = *p* < 0.0001. **B.** Relative expression thresholds that maximize both sensitivity and specificity for each genes.

The use of qPCR assays validated significant changes in gene expression level of CYP1A2, CYP2B6, CYP2C19, CYP26A1, CYP17A1, and CYP19A1 in HCC tissues initially identified by RNA-Seq. These genes were then selected to evaluate their potential to discriminate between HCC and normal samples. For this, receiver operating characteristics (ROC) curves presenting the sensitivity (true positive rate) and specificity (true negative rate) at each expression level threshold were drawn, and the area under the curve (AUC) was determined for each gene. A value of AUC close to 1 indicates that the test classifies the samples as normal or cancer correctly, while an AUC of 0.5 indicates no predictive power. Then, an expression threshold that maximizes both sensitivity and specificity was identified for each gene (Figure 5A). These parameters are both important in this type of test since a high sensitivity allows the detection of all pathologic samples while a high specificity assures a negative result in normal samples. A gene with a AUC of at least 0.95, and sensitivity and specificity of 90% or more at the established threshold was considered adequate for confident identification of HCC tissues. Using these criteria, 3 repressed CYP450 were selected: CYP2B6 (AUC: 0.9821, sensitivity: 90.5%, specificity: 100%), CYP1A2, and CYP2C19, with the last 2 genes classifying with AUC: 1, sensitivity: 100%, specificity: 100% (Figure 5B).

### Protein level of potential biomarkers in HCC and their fold change across cancers

**Figure 6:**
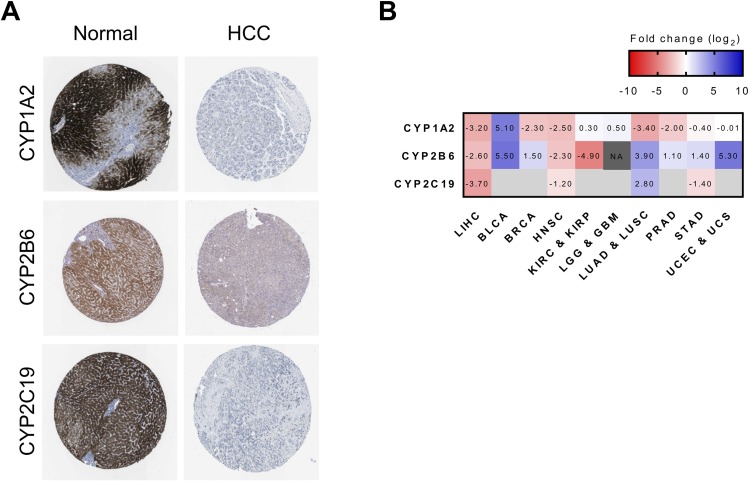
Investigation of potential biomarkers at protein levels in HCC and expression fold-change across cancers **A.** Protein expression of potential biomarkers genes CYP1A2, CYP2B6 and CYP2C19, detected by immunohistochemistry, in normal liver and HCC tissues. These images were extracted from the Human Protein Atlas database, according to its academic usage permission (www.proteinatlas.org) [47]. **B.** Fold-change of CYP1A2, CYP2B6, and CYP2C19 expression (log_2_ gene expression, in FPKM-UQ, fold change) in different cancers (liver hepatocellular carcinoma, urothelial bladder carcinoma, breast invasive carcinoma, head and neck squamous cell carcinoma, kidney renal clear cell carcinoma and kidney renal papillary cell carcinoma, brain lower grade glioma and glioblastoma multiforme, lung adenocarcinoma and lung squamous cell carcinoma, prostate adenocarcinoma, stomach adenocarcinoma, uterine corpus endometrial carcinoma and uterine carcinosarcoma). Number of normal and cancer samples are presented in figure S3B. Overexpression levels are shown in blue and repression levels in red. Grey positions are missing data, and NA indicates the impossibility to determine a fold change because the gene was not detected in normal samples.

The expression of CYP1A2, CYP2B6, and CYP2C19 at mRNA level was shown to discriminate HCC from normal samples, but, ultimately, a screening test based on the protein expression level of these genes would be preferred for clinical application. To evaluate this possibility, the protein expression levels of the selected candidates were investigated in healthy and HCC liver tissues using immunohistochemistry data of The Human Protein Atlas. Each gene was detected by a specific antibody coupled to a horseradish peroxidase detection system. These data showed that expression of these 3 genes is repressed at the protein level in cancer tissues since the quantity and intensity of the coloration can be positively correlated with the expression level of the protein (Figure 6A).

Finally, to evaluate if the dysregulation of these three selected genes is unique to HCC or could be found in other cancers, fold-changes were calculated in different types of tumors compared to their normal tissues (number of samples are shown in Supplementary Figure 3B). RNA-Seq data, obtained from the National Cancer Institute Genomic Data Commons (GDC) Data Portal, from liver, bladder, breast, head and neck, kidney, lung, prostate, stomach, uterine, and brain tissues were then compared. It should be noted that this database quantifies mRNA expression levels in FPKM-UQ rather than TPM, which can cause minor variations in fold-changes calculated previously with CYP450 genes in liver cancer. This analysis showed that each of these cancer types has a distinct pattern of expression for CYP1A2, CYP2B6, and CYP2C19 (Figure 6B). Moreover, a global look at expression variation of the 57 CYP450 genes across these 10 cancers showed a similar trend, indicating that every cancer type has a specific signature in regards to the expression of CYP450 genes (Supplementary Figure 3C). Nevertheless, it is possible to see that CYP2W1 is overexpressed in all cancers, except in prostate adenocarcinoma (PRAD). As mentioned previously in others studies, this particular CYP450 could be used as a biomarker to screen for tumor presence and potentially in treatments since it can activate pro-drugs only when it is expressed in cancer cells [20–22]. Note that this gene was excluded of potential biomarkers for HCC in this study since the up-regulation in paired samples was not statistically significant (*p* = 0.500) and that the expression level in the samples was inferior to 2 TPM. A similar pattern can be observed for CYP4F8, which is up-regulated in all cancers, except PRAD and head and neck squamous cell carcinoma (HNSC).

## DISCUSSION

Advances in genomic technologies have allowed the characterization of molecular events occurring during carcinogenesis, such as mRNA expression dysregulation, which could potentially lead to the identification of new biomarkers. In the case of HCC, differential gene expression levels of various CYP450s have previously been reported [23–26]. However, these previous studies were generally performed on a limited number of HCC tissue samples or focused on a small subset on CYP450s. In contrast, in the present study focusing on the entire CYP450 family (57 CYP450 genes), we monitored the expression landscape of CYP450s in more than 200 HCC tissues using RNA-Seq data from The Cancer Genome Atlas. Following rigorous statistical and validation assays, 3 potential biomarkers for HCC were identified: CYP1A2, CYP2B6, and CYP2C19. Our analysis also demonstrated that the observed change of expression for these three genes is relatively constant from stage I to stage IV, suggesting that the identified CYP450s could be suitable biomarkers for early identification of HCC.

Various members of the CYP450s family were previously identified as interesting for cancer screening or treatments. For instance, CYP2J2 and CYP2W1 were both found to have a higher level of expression in carcinoma cells and transformed tissues where they could have a role in the progression or treatment of cancers [20, 27]. CYP1B1 is one of the best-known CYP450s up-regulated in multiple cancers, like breast, colon and brain cancer [28], and studies are in progress to use it as a therapeutic target in the treatment of cancers [29]. Moreover, CYP17A1 has also been extensively characterized, both at the mRNA and protein levels, in tissues and sera of HCC patients. A previous study showed that a threshold of 60.2ng/mL allows identification of HCC patients with a sensitivity and specificity of 86.9% and 76.8% respectively, while a combination with AFP achieved 90.1% and 80.3% of sensitivity and specificity [30].

In addition to the identification of CYP450s as markers for HCC, the roles of CYP450s in carcinogenesis merits further investigation. Indeed, CYP1A2, CYP2B6, and CYP2C19, which are repressed in HCC, are involved in the metabolism of eicosanoids, drugs, and foreign chemicals [17]. This could potentially promote HCC development by an accumulation of toxic compounds for cells. Interestingly, CYP1A2 is the major CYP450 found in the liver and is involved in the metabolism of 8.9% of drugs used in the clinic, while CYP2B6 and CYP2C19 are involved in the metabolism of 7.2% and 6.8%, respectively of these drugs [31]. The lower levels of these enzymes in HCC patients could then have an impact on their susceptibility to drugs doses or the activation of pro-drugs. Similarly, CYP1B1 is generally absent from normal adult liver and its expression is associated with carcinogenesis, partly because it can activate pro-carcinogens [32]. On the other hand, CYP26A1, which is repressed in HCC, is involved in retinoic acid inactivation [17]. It is therefore possible that products, such as retinoic acid, would have a role in the progression of HCC since a previous study revealed that vitamin A deficiency, which could result from a CYP26A1 depletion, is associated with increased susceptibility to carcinogenesis [33]. Finally, in hormone-dependent prostate and breast cancers, CYP17A1 and CYP19A1 are targeted by inhibitors for cancer treatments [34, 35]. In the case of HCC, different studies showed that a high level of estrogens would be protective for patients, which can correlate with the fact that HCC is much more present in men than women [36, 37].

Several studies are seeking the identification of new HCC biomarkers. Some of these potential markers such as AFP lectin-bound (AFP-L3) [38], Des-γ-carboxy prothrombin [39], or glypican-3 (GPC3) [40], have interesting diagnosis performances. Despite a large number of promising molecules, individual markers generally lack sensitivity and/or specificity to be sufficiently effective. The future of HCC screening will most likely involve the use of a combination of biomarkers based on various macromolecules such as mRNAs, proteins, mi-RNAs, or even powerful imagery techniques such as ultrasonography.

## MATERIALS AND METHODS

### RNA-Seq data

RNA-Seq expression data files were obtained from the National Cancer Institute Genomic Data Commons (GDC) Data Portal (https://portal.gdc.cancer.gov). Expression data was imported from two analysis format: transcripts per million (TPM) and upper quartile fragments per kilobase of transcript per million mapped reads (FPKM-UQ). TPM data was derived by read alignement using Burrows-Wheeler Aligner (http://bio-bwa.sourceforge.net) on GRCh37/hg19 reference genome, and quantification using RSEM (https://deweylab.github.io/RSEM). TPM data at the GDC is available as legacy archive (https://portal.gdc.cancer.gov/legacy-archive) as it was originally obtained from The Cancer Genome Atlas (TCGA) data portal, now superceded by GDC. FPKM-UQ data workflow involves read alignement on GRCh38 reference genome using STAR (https://github.com/alexdobin/STAR) and quantification following the Genomic Data Commons workflow (https://docs.gdc.http://cancer.gov/Data/Bioinformatics_Pipelines/Expression_mRNA_Pipeline).

### Gene expression analysis

Analyses were performed on the transcriptomic data of 220 hepatocellular carcinoma (HCC) and 38 normal liver tissues, uninfected by hepatitis B virus and/or hepatitis C virus, generated by The Cancer Genome Atlas (http://cancergenome.nih.gov/). The list, containing the 23,393 detected genes, was filtered to keep only data with at least two replicates for both normal and tumor samples. Fold changes between average transcripts per million (TPM) of tumor tissues compared to normal tissues and q-value were calculated. A positive fold change value indicates that the gene is overexpressed in HCC while a negative value indicates its repression. Data for which the q-value were under 0.05 and fold change in base 2 logarithm equal or higher than 2 in absolute value were considered significant and were kept (4,205 genes). For cytochromes P450, similar criteria were used, and a cut-off of a mean expression of at least 2 TPM in one or the other condition was added to allow efficient detection of the transcript in samples.

### Gene ontology analysis

Enriched biological processes in filtered gene list were determined by using the database for annotation, visualization and integrated discovery [41, 42] (DAVID, V6.7, https://david.ncifcrf.gov/). All 23,393 detected genes were used as background and 75 repressed genes were analyzed.

### String networks

The list of 75 repressed genes was submitted to the STRING database [43] (Search Tool for the Retrieval of Interacting Genes, version 10.0, www.string-db.org) to produce a protein-protein interactions network, from the *Homo sapiens* interactome.

### Determination of target genes expression by qPCR on cDNA samples

TissueScan Liver Cancer cDNA Arrays from OriGene Technologies (cat. LVRT301; Rockville, MD, USA) were assessed for the expression of dysregulated cytochromes P450 using the manufacturer's protocol. The plates contained cDNAs from 8 normal and 22 hepatocellular carcinomas tissues, from which 6 normal and HCC samples are paired, and were analyzed by quantitative PCR (qPCR). All forward and reverse primers were individually resuspended to 20-100 μM in Tris-EDTA buffer (IDT) and diluted as a primer pair to 1 μM in RNase DNase-free water (IDT). Primer design and validation were evaluated as described previously [44] and the amplified products were analyzed by automated chip-based microcapillary electrophoresis on Labchip GX Touch HT instruments (Perkin Elmer). qPCR reactions were performed in 10 μl in 384 well plates on a CFX-384 thermocycler (BioRad) with 5 μL of 2X iTaq Universal SYBR Green Supermix (BioRad), 10 ng (3 μl) cDNA, and 200 nM final (2 μl) primer pair solutions. The following cycling conditions were used: 3 min at 95°C; 50 cycles: 15 sec at 95°C, 30 sec at 60°C, 30 sec at 72°C. Relative expression levels were calculated using the qBASE framework [45] and the housekeeping genes YWHAZ, MRPL19 and SDHA for human cDNA. For every PCR run, control reactions performed in the absence of template were performed for each primer pair and these were consistently negative. Amplicon sizing and relative quantitation were performed by the manufacturer's software. cDNA samples and patients information are available at www.origene.com.

### ROC curve analysis

Receiver operating characteristics (ROC) curves, and associated area under the curve (AUC), were generated from qPCR relative expression data of significantly dysregulated CYP450, which are CYP17A1, CYP19A1, CYP1A2, CYP2B6, CYP2C19 and CYP26A1, to evaluate their capacity to distinguish normal from HCC samples. Expression thresholds that maximize both sensitivity and specificity and the associated characteristics were determined for each gene.

### Immunohistochemistry

The proteins of interest were detected by immunohistochemistry using specific antibodies and horseradish peroxidase detection, in healthy liver and HCC tissues. These images were extracted from The Human Protein Atlas [46], according to its academic usage permission (Data and complete protocol can be found at www.proteinatlas.org).

### Statistical analysis

GraphPad Prism (version 7.03) was used for statistical analysis and ROC curves. Data are presented as mean ± standard deviation. The distribution shape of the data was analyzed by Shapiro-Wilk test of normality. Mann-Whitney U test was used to compare two distribution groups and Wilcoxon rank sum test for paired samples. Student's *t*-test was used for qPCR analysis of paired samples. P value and q values less than 0.05 (two-tailed) were considered significant. ^*^ = *p* < 0.05; ^**^ = *p* < 0.01; ^***^ = *p* < 0.001; ^****^ = *p* < 0.0001.

## SUPPLEMENTARY FIGURES AND TABLE



## Abbreviations

AFP: α-fetoprotein
AUC: Area under the curve
cDNA: complementary DNA
CYP450: Cytochrome P450
FPKM-UQ: Upper quartile fragments per kilobase of transcript per million
HBV: Hepatitis B virus
HBCV: Hepatitis B and C virus
HCC: Hepatocellular carcinoma
HCV: Hepatitis C virus
NNoHV: Normal tissue, no hepatitis virus
ROC curves: Receiver operating characteristics
TCGA: The Cancer Genome Atlas
TNoHV: Tumor tissue, no hepatitis virus
TPM: Transcript per million

## References

[1] Ferlay J, Soerjomataram I, Dikshit R, Eser S, Mathers C, Rebelo M (2015). Cancer incidence and mortality worldwide: Sources, methods and major patterns in GLOBOCAN 2012. Int J Cancer.

[2] Han Z-G (2012). Functional genomic studies: insights into the pathogenesis of liver cancer.

[3] The Cancer Genome Atlas Research Network (2017). Comprehensive and integrative genomic characterization of hepatocellular carcinoma. Cell.

[4] American Cancer Society (2016). Cancer Facts & Figures 2016. Atlanta Am Cancer Soc.

[5] Lin S, Hoffmann K, Schemmer P (2012). Treatment of hepatocellular carcinoma: A systematic review. Liver cancer.

[6] Bialecki ES, Di Bisceglie AM (2005). Diagnosis of hepatocellular carcinoma. HPB.

[7] Lou J, Zhang L, Lv S, Zhang C, Jiang S (2012). Biomarkers for hepatocellular carcinoma. Clin J Gastroenterol.

[8] Robinson PJA (2000). Imaging liver metastases: current limitations and future prospects. Br J Radiol.

[9] Sauzay C, Petit A, Bourgeois AM, Barbare JC, Chauffert B, Galmiche A (2016). Alpha-foetoprotein (AFP): A multi-purpose marker in hepatocellular carcinoma. Clin Chim Acta.

[10] Liu C, Xiao GQ, Yan LN, Li B, Jiang L, Wen TF (2013). Value of α-fetoprotein in association with clinicopathological features of hepatocellular carcinoma. World J Gastroenterol.

[11] Peng SY, Chen WJ, Lai PL, Jeng YM, Sheu JC, Hsu HC (2004). High α-fetoprotein level correlates with high stage, early recurrence and poor prognosis of hepatocellular carcinoma: Significance of hepatitis virus infection, age, p53 and β-catenin mutations. Int J Cancer.

[12] Lu Y, Zhu M, Li W, Lin B, Dong X, Chen Y (2016). Alpha fetoprotein plays a critical role in promoting metastasis of hepatocellular carcinoma cells. J Cell Mol Med.

[13] Colombo M (2001). Screening for cancer in viral hepatitis. Clin Liver Dis.

[14] O'Conor GT, Tatarinov YS, Abelev G, Uriel J (1970). A collaborative study for the evaluation of a serologic test for primary liver cancer. Cancer.

[15] Kondo Y, Kimura O, Shimosegawa T (2015). Significant biomarkers for the management of hepatocellular carcinoma. Clin J Gastroenterol.

[16] Zhang C, Peng L, Zhang Y, Liu Z, Li W, Chen S (2017). The identification of key genes and pathways in hepatocellular carcinoma by bioinformatics analysis of high-throughput data. Med Oncol.

[17] Nebert DW, Russell DW (2002). Clinical importance of the cytochromes P450. Lancet.

[18] Nishimura M, Yaguti H, Yoshitsugu H, Naito S, Satoh T (2003). Tissue distribution of mRNA expression of human cytochrome P450 isoforms assessed by high-sensitivity real-time reverse transcription PCR. Yakugaku Zasshi.

[19] Nebert DW, Wikvall K, Miller WL (2013). Human cytochromes P450 in health and disease. Philos Trans R Soc Lond B Biol Sci.

[20] Karlgren M, Gomez A, Stark K, Svärd J, Rodriguez-Antona C, Oliw E (2006). Tumor-specific expression of the novel cytochrome P450 enzyme, CYP2W1. Biochem Biophys Res Commun.

[21] Nishida CR, Lee M, de Montellano PRO (2010). Efficient hypoxic activation of the anticancer agent AQ4N by CYP2S1 and CYP2W1. Mol Pharmacol.

[22] Pan Y, Ong EC (2017). Cytochrome P450 2W1 (CYP2W1) - ready for use as the biomarker and drug target for cancer?. Xenobiotica.

[23] Yan T, Lu L, Xie C, Chen J, Peng X, Zhu L (2015). Severely impaired and dysregulated cytochrome P450 expression and activities in hepatocellular carcinoma: Implications for personalized treatment in patients. Mol Cancer Ther.

[24] Wai-Hung Ho D, Ka-Lun Kai A, Oi-Lin Ng I (2015). TCGA whole-transcriptome sequencing data reveals significantly dysregulated genes and signaling pathways in hepatocellular carcinoma. Front Med.

[25] Huang Q, Lin B, Liu H, Ma X, Mo F, Yu W (2011). RNA-seq analyses generate comprehensive transcriptomic landscape and reveal complex transcript patterns in hepatocellular carcinoma. PLoS One.

[26] Fan W, Ye G (2018). Microarray analysis for the identification of specific proteins and functional modules involved in the process of hepatocellular carcinoma originating from cirrhotic liver. Mol Med Rep.

[27] Jiang JG, Chen CL, Card JW, Yang S, Chen JX, Fu XN (2005). Cytochrome P450 2J2 promotes the neoplastic phenotype of carcinoma cells and is up-regulated in human tumors. Cancer Res.

[28] Murray GI, Taylor MC, McFadyen MCE, McKay JA, Greenlee WF, Burke MD (1997). Tumor-specific expression of cytochrome P450 CYP1B1. Cancer Res.

[29] Gribben JG, Ryan DP, Boyajian R, Urban RG, Hedley ML, Beach K (2005). Unexpected association between induction of immunity to the universal tumor antigen CYP1B1 and response to next therapy. Clin Cancer Res.

[30] Wang F, Huang J, Zhu Z, Ma X, Cao L, Zhang Y (2016). Transcriptome analysis of WHV/c-myc transgenic mice implicates CYP17A1 as a promising biomarker for hepatocellular carcinoma. Cancer Prev Res.

[31] Zanger UM, Schwab M (2013). Cytochrome P450 enzymes in drug metabolism: Regulation of gene expression, enzyme activities, and impact of genetic variation. Pharmacol Ther.

[32] Shimada T, Hayes CL, Yamazaki H, Amin S, Hecht SS, Guengerich FP (1996). Activation of chemically diverse procarcinogens by human cytochrome P-450 IBI. Cancer Res.

[33] Lotan R (1991). Retinoids as modulators of tumor cells invasion and metastasis. Semin Cancer Biol.

[34] Vasaitis TS, Bruno RD, Njar VCO (2011). CYP17 inhibitors for prostate cancer therapy. J Steroid Biochem Mol Biol.

[35] Del Re M, Michelucci A, Simi P, Danesi R (2012). Pharmacogenetics of anti-estrogen treatment of breast cancer. Cancer Treat Rev.

[36] Xu H, Wei Y, Zhang Y, Xu Y, Li F, Liu J (2012). Estrogen attenuates tumour progression in hepatocellular carcinoma. J Pathol.

[37] Torre LA, Bray F, Siegel RL, Ferlay J, Lortet-tieulent J, Jemal A (2015). Global Cancer Statistics, 2012. CA a cancer J Clin.

[38] Taketa K, Sekiya C, Namiki M, Akamatsu K, Ya Ohta, Endo Y (1990). Lectin-reactive profiles of alpha-fetoprotein characterizing hepatocellular carcinoma and related conditions. Gastroenterology.

[39] Liebman HA, Furie BC, Tong MJ, Blanchard RA, Lo J, Lee S-D (1984). Des-γ-Carboxy (Abnormal) Prothrombin as a Serum Marker of Primary Hepatocellular Carcinoma. N Engl J Med.

[40] Zhu ZW, Friess H, Wang L, Abou-Shady M, Zimmermann A, Lander AD (2001). Enhanced glypican-3 expression differentiates the majority of hepatocellular carcinomas from benign hepatic disorders. Gut.

[41] Huang DW, Sherman BT, Lempicki RA (2009). Systematic and integrative analysis of large gene lists using DAVID bioinformatics resources. Nat Protoc.

[42] Huang DW, Sherman BT, Lempicki RA (2009). Bioinformatics enrichment tools: Paths toward the comprehensive functional analysis of large gene lists. Nucleic Acids Res.

[43] Szklarczyk D, Franceschini A, Wyder S, Forslund K, Heller D, Huerta-Cepas J (2015). STRING v10: Protein-protein interaction networks, integrated over the tree of life. Nucleic Acids Res.

[44] Brosseau JP, Lucier JF, Lapointe E, Durand M, Gendron D, Gervais-Bird J (2010). High-throughput quantification of splicing isoforms. RNA.

[45] Hellemans J, Mortier G, De Paepe A, Speleman F, Vandesompele J (2007). qBase relative quantification framework and software for management and automated analysis of real-time quantitative PCR data. Genome Biol.

[46] Uhlén M, Björling E, Agaton C, Szigyarto CA-K, Amini B, Andersen E (2005). A human protein atlas for normal and cancer tissues based on antibody proteomics. Mol Cell Proteomics.

[47] Berglund L, Björling E, Oksvold P, Fagerberg L, Asplund A, Szigyarto CA (2008). A genecentric Human Protein Atlas for expression profiles based on antibodies. Mol Cell Proteomics.

