# Supplementary material for: Dissecting the expression landscape of cytochromes P450 in hepatocellular carcinoma: towards novel molecular biomarkers
Source: Genes Cancer. 2019 May;10(3-4):97–108. doi: 10.18632/genesandcancer.190 (PMC6584210; doi:10.18632/genesandcancer.190)
Supplement: Supplementary file 1 [file ganc-10-097-s001.pdf]

Dissecting the expression landscape of cytochromes P450 in hepatocellular carcinoma: towards novel molecular biomarkers – Brodeur et al

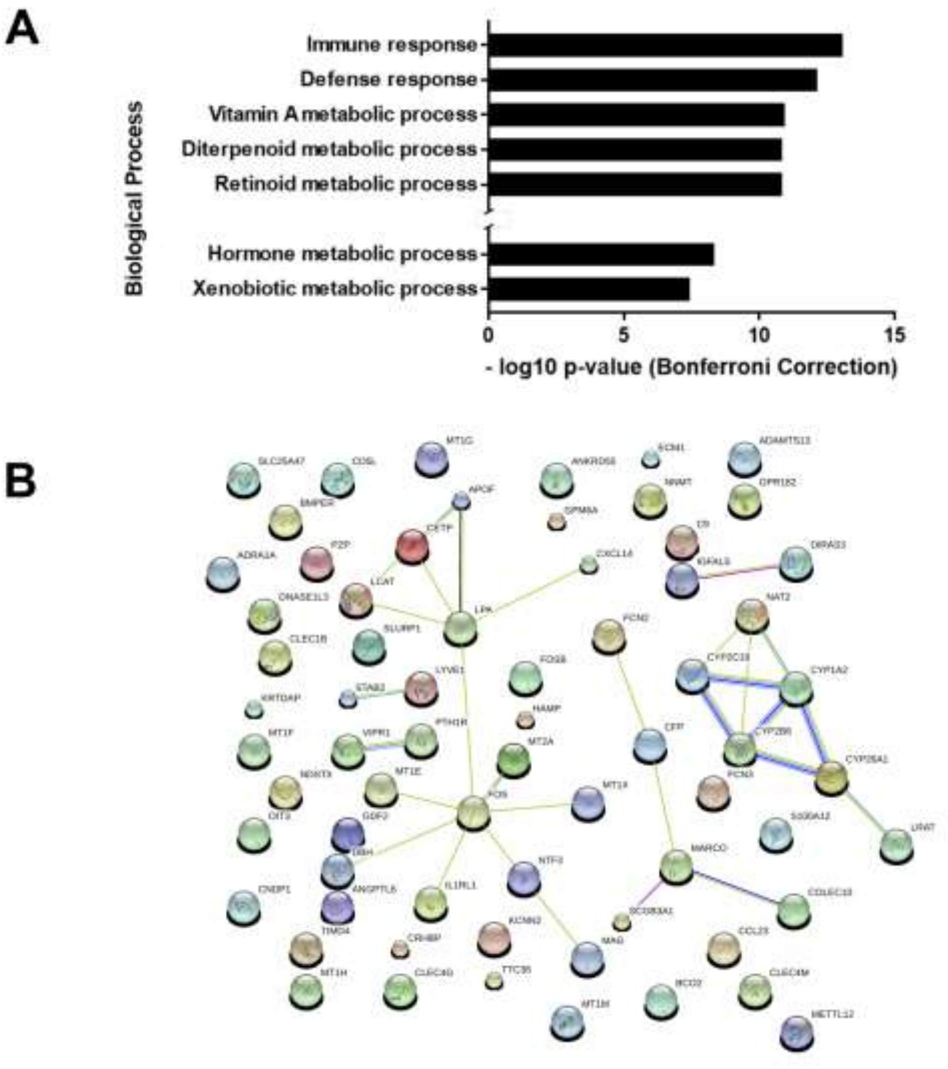

Figure S1: Gene ontology and protein-protein interaction analysis of repressed genes in HCC. (A) Gene ontology analysis of the 75 repressed genes in HCC. Enriched biological processes (BP) were determined using DAVID. The top five most enriched BP are presented as well as two other BPs significantly enriched, encomassing cytochromes P450s. (B) Complete protein-protein interaction network of the 75 repressed genes in HCC. The network was determined by uploading the gene list into STRING [43].

A

	NoHV	HBV	HCV	HBCV	Total
Normal	38	7	5	0	50
Tumor	220	96	48	7	371

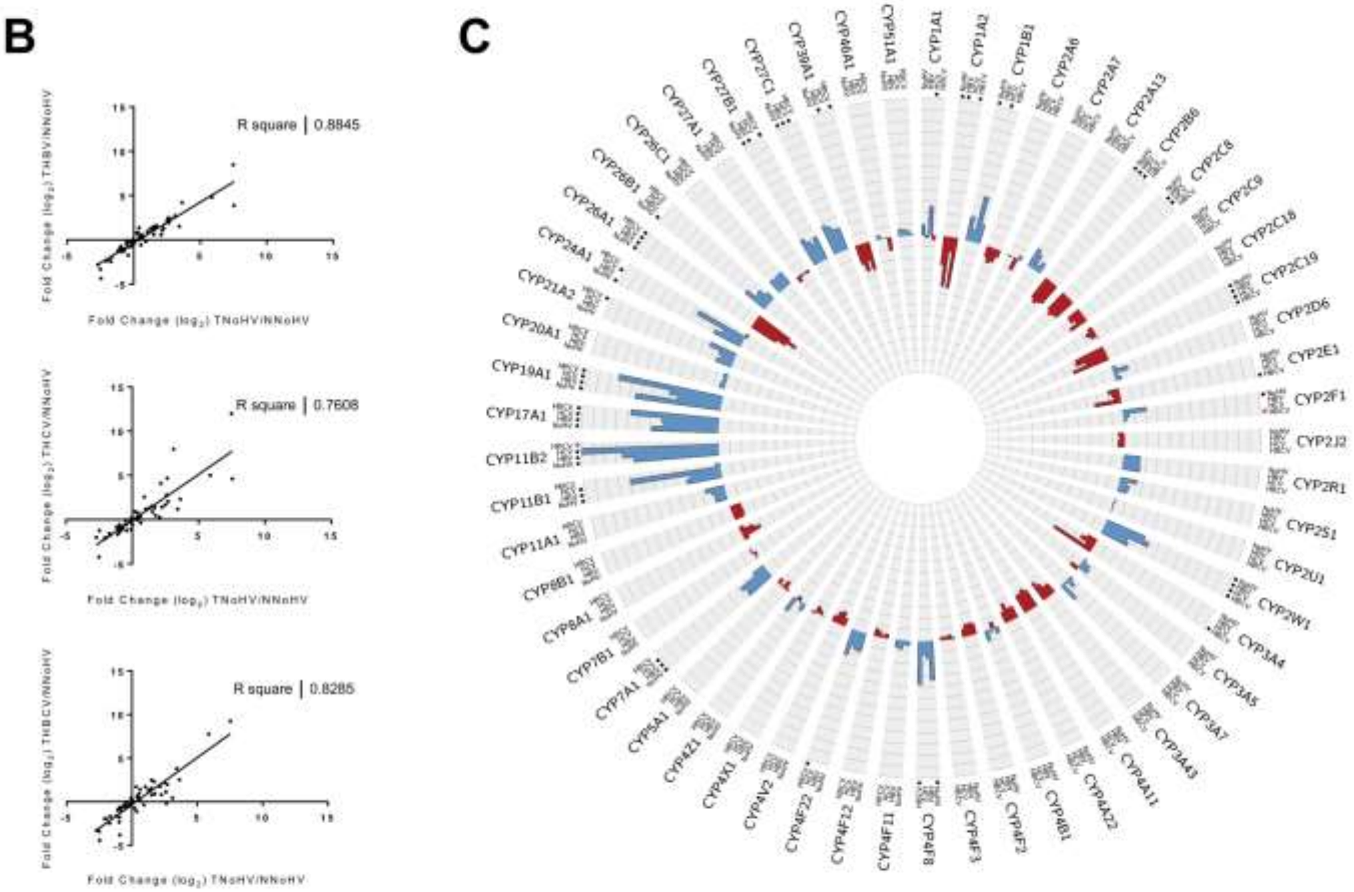

Figure S2: Expression variation profiles of cytochromes P450 in HCC. (A) Distribution of non-infected (NoHV) and hepatitis B virus- (HBV), hepatitis C virus- (HCV) or hepatitis B and C viruses-infected (HBCV) normal and tumor tissues samples. (B) Correlation between fold change in HBV-, HCV-, or HBCV- and non-infected tumors, all compared to non-infected normal tissues. (C) Comparison of 57 CYP450 expression fold-change, compared to non-infected normal tissues, in non-infected, HBV-, HCV- and HBCV-infected tumor tissues. Overexpression is shown in blue and repression in red, on a scale from -12 to 12. Black dots indicate a fold change of 4 or more and pink stars indicate unavailable data.

A

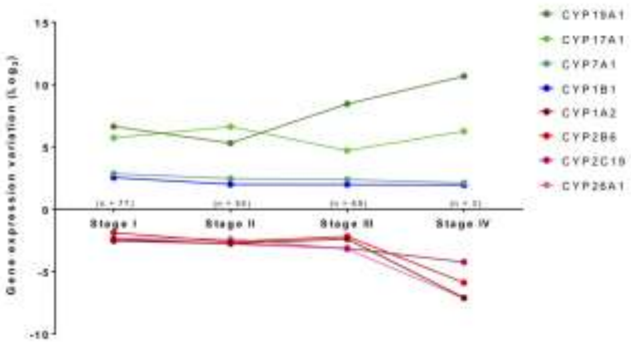

B

Cancer	Normal	Tumor
LIHC	50	374
BLCA	19	411
BRCA	13	1097
HNSC	44	500
KIRC & KIRP	104	822
LGG & GBM	5	695
LUAD & LUSC	108	1027
PRAD	52	498
STAD	32	375
UCEC & UCS	35	604

C

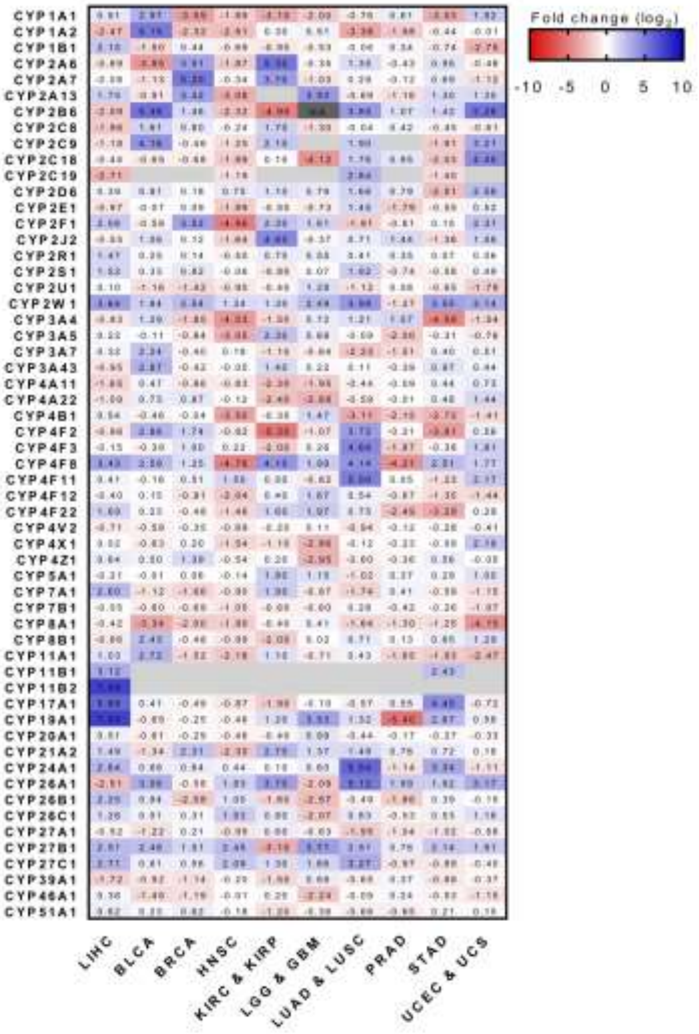

Figure S3: Investigation of cytochromes P450 characteristics across HCC stage or cancers. (A) Fold change expression of the eight dysregulated CYP450 in hepatocellular carcinoma relative to their expression in normal tissues (expression in base 2 logarithm of the ratio of mean expression, in transcript-per-million, of tumor tissues on mean expression in normal tissues, both uninfected by hepatitis virus) according to the pathologic stage of the tumor. (B) Number of normal and cancer samples used to determine expression variation of CYP450 in 12 cancers, according to the TCGA RNA-seq data. (LIHC: liver hepatocellular carcinoma, BLCA: urothelial bladder carcinoma, BRCA: breast invasive carcinoma, HNSC: head and neck squamous cell carcinoma, KIRC: kidney renal clear cell carcinoma, KIRP: kidney renal papillary cell carcinoma, LGG: brain lower grade glioma, GBM: glioblastoma multiforme, LUAD: lung adenocarcinoma, LUSC: lung squamous cell carcinoma, PRAD: prostate adenocarcinoma, STAD: stomach adenocarcinoma, UCEC: uterine corpus endometrial carcinoma, UCS: uterine carcinosarcoma). (C) Fold-change of the 57 CYP450 expression (log_2 gene expression, in FPKM-UQ, fold-change in tumor compared to normal tissues) in different cancers.

Overexpression is shown in blue and repression in red. Grey positions are missing data, and NA indicates the impossibility to determine a fold change because the gene was not detected in normal samples.

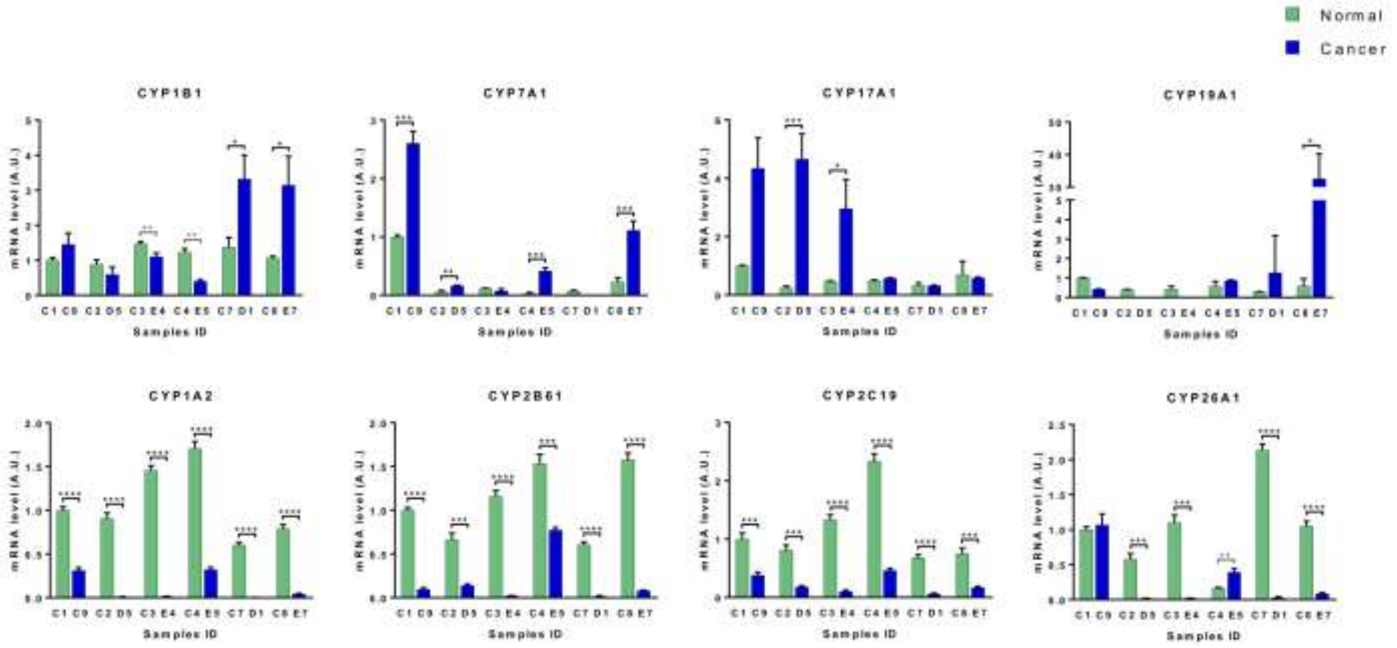

Figure S4: Paired samples analysis of the cDNA samples used for validation of cytochromes P450 dysregulated in HCC. Analysis of the six paired tumor (blue) and normal (green) cDNA samples used for expression validation of cytochromes P450 by qPCR. Mean expression and standard deviation values for each sample were obtained from three technical replicates and are plotted in arbitrary units. Global analysis of the eight normal and 22 tumor samples are available in figure 4. Student *t*-test. *=p<0.05; **=p<0.01; ***=p<0.001; ****=p<0.0001.

	TNoHV/NNoHV		THBV/NNoHV		THCV/NNoHV		THBCV/NNoHV	
	Log_2Ratio	q-value	Log_2Ratio	q-value	Log_2Ratio	q-value	Log_2Ratio	q-value
CYP1A1	0,91	1,1E-02	-0,18	8,5E-02	2,55	7,5E-03	-0,38	1,7E-01
CYP1A2	-2,47	1,7E-09	-4,36	1,5E-10	-1,25	5,2E-04	-4,44	5,9E-08
CYP1B1	2,15	4,3E-05	1,05	3,8E-04	4,11	3,7E-02	1,72	8,0E-02
CYP2A6	-0,89	8,3E-06	-1,36	1,8E-07	-1,17	1,6E-05	-0,66	9,6E-02
CYP2A7	-0,06	8,6E-02	0,02	1,3E-01	-1,10	9,0E-03	0,39	1,9E-01
CYP2A13	1,75	3,2E-03	1,41	2,3E-02	0,46	7,7E-02	0,92	1,4E-01
CYP2B6	-2,09	5,9E-08	-2,38	4,9E-08	-2,15	3,8E-07	-1,97	1,4E-02
CYP2C8	-1,96	6,8E-14	-2,27	3,0E-14	-2,03	1,9E-12	-1,94	8,1E-05
CYP2C9	-1,16	2,2E-13	-1,27	3,8E-13	-0,87	5,7E-06	-1,65	3,0E-03
CYP2C18	-0,44	1,7E-04	-0,82	1,5E-07	-0,54	4,1E-04	-0,01	2,3E-01
CYP2C19	-2,71	7,3E-06	-2,81	1,4E-05	-2,01	3,0E-04	-3,30	8,8E-04
CYP2D6	0,39	5,6E-03	1,36	3,0E-09	0,26	7,0E-02	0,53	1,2E-01
CYP2E1	-0,97	2,7E-07	-0,72	1,7E-04	-0,56	3,4E-03	-2,34	1,3E-02
CYP2F1	2,06	8,8E-03	0,65	6,6E-03	0,28	2,5E-02		
CYP2J2	-0,55	8,3E-10	-0,43	4,0E-05	-0,56	7,9E-07	-0,56	4,5E-02
CYP2R1	1,47	4,2E-37	1,47	5,0E-20	1,49	7,0E-12	1,54	2,9E-03
CYP2S1	1,52	3,0E-04	0,61	9,0E-03	0,90	2,2E-02	0,78	9,9E-02
CYP2U1	0,10	3,0E-02	-0,06	9,4E-02	0,05	1,0E-01	0,02	2,2E-01
CYP2W1	3,64	4,4E-05	4,20	6,8E-03	2,31	1,7E-05	2,52	4,8E-02
CYP3A4	-0,93	2,5E-05	-1,39	2,6E-06	-0,69	1,2E-02	-3,82	7,0E-09
CYP3A5	0,22	3,7E-02	0,78	1,3E-03	0,91	1,2E-02	-0,74	6,5E-02
CYP3A7	0,32	3,4E-02	0,82	1,1E-02	0,17	1,2E-01	1,70	7,6E-02
CYP3A43	-0,95	2,1E-06	-0,94	1,8E-04	-1,39	6,0E-06	-1,81	8,1E-03
CYP4A11	-1,65	1,9E-16	-1,92	3,3E-19	-1,74	1,3E-15	-1,68	1,0E-03
CYP4A22	-1,09	1,3E-05	-1,10	3,6E-05	-1,67	1,8E-06	-0,98	4,1E-02
CYP4B1	0,54	2,6E-02	-0,57	4,9E-02	0,31	8,8E-02	0,92	1,4E-01
CYP4F2	-0,96	2,7E-10	-1,08	3,0E-10	-1,06	2,5E-09	-0,68	4,5E-02
CYP4F3	-0,15	1,5E-02	-0,39	5,7E-04	-0,25	2,2E-02	-0,30	1,4E-01
CYP4F8	3,43	2,4E-03	1,52	3,8E-03	1,19	1,9E-02	3,80	
CYP4F11	0,41	7,9E-06	0,41	1,8E-03	0,75	5,1E-04	0,63	7,6E-02
CYP4F12	-0,40	1,6E-04	-0,19	4,0E-02	-0,52	3,7E-04	-0,67	4,4E-02
CYP4F22	1,69	4,5E-10	1,61	1,7E-04	1,35	8,4E-03	2,30	3,3E-02
CYP4V2	-0,71	2,9E-08	-1,21	3,8E-12	-1,04	4,9E-10	-0,57	2,7E-02
CYP4X1	0,02	7,1E-02	-0,38	2,6E-02	-0,57	3,8E-03	0,21	1,8E-01
CYP4Z1	0,64	8,8E-03	0,14	9,5E-02	-0,36	4,3E-02	1,40	1,3E-01
CYP5A1	-0,21	2,7E-02	-0,74	5,9E-04	-0,08	1,1E-01	-0,47	7,9E-02
CYP7A1	2,60	1,3E-13	2,46	1,3E-06	2,79	2,3E-04	1,13	4,2E-02
CYP7B1	-0,05	5,6E-02	-0,25	1,9E-02	0,21	4,8E-02	-0,02	2,2E-01
CYP8A1	-0,42	1,5E-02	-1,74	2,7E-08	-1,23	4,1E-05	-0,41	1,7E-01
CYP8B1	-0,86	1,2E-08	-1,00	7,7E-08	-0,91	3,8E-05	-0,94	5,0E-02
CYP11A1	1,03	4,6E-07	0,92	9,4E-05	1,20	2,7E-05	1,70	7,5E-02
CYP11B1	3,12		2,70		7,98		0,44	
CYP11B2	7,48	2,3E-02	6,47	5,3E-02	11,99	5,4E-02		
CYP17A1	5,88	2,1E-09	4,81	1,3E-04	5,01	3,4E-03	7,78	1,1E-01
CYP19A1	7,54	2,0E-04	3,85	5,7E-05	4,63	2,1E-03	9,25	1,2E-01
CYP20A1	0,51	1,1E-10	0,38	8,4E-06	0,28	2,8E-03	0,40	1,1E-01
CYP21A2	1,49	9,8E-12	1,16	4,2E-06	1,46	3,1E-04	2,48	5,2E-02
CYP24A1	2,84	3,5E-03	1,93	3,6E-02	4,67	3,4E-02	-0,21	
CYP26A1	-2,51	2,2E-04	-3,33	1,9E-04	-4,23	1,5E-04	-3,42	8,1E-03
CYP26B1	2,25	3,0E-05	1,32	2,2E-03	1,43	1,8E-02	0,79	1,3E-01
CYP26C1	1,26	3,2E-08	1,22	1,9E-03	1,27	2,8E-02	1,31	9,6E-02
CYP27A1	-0,52	4,0E-09	-0,16	1,3E-02	-0,04	1,0E-01	-0,24	1,4E-01
CYP27B1	2,51	1,5E-10	2,10	1,4E-05	1,88	2,3E-04	2,17	4,9E-02
CYP27C1	2,71	3,4E-12	2,42	1,9E-09	2,05	1,2E-03	1,97	4,5E-02
CYP39A1	-1,72	3,6E-18	-2,46	2,9E-20	-1,58	1,3E-12	-2,47	1,6E-03
CYP46A1	0,36	5,0E-03	0,01	1,3E-01	-0,02	1,2E-01	-1,13	7,3E-03
CYP51A1	0,62	5,5E-04	0,57	1,9E-03	0,44	1,7E-02	0,10	1,9E-01

Table S1. List of cytochromes P450 fold change expression in non-infected or HBV, HCV, and HBCV infected HCC compared to normal non-infected liver tissues. Fold-change (in log_2 gene expression, in TPM, fold change) and associated q-value of gene expression variation of tumor tissues compared to normal ones are shown for the 57 cytochromes P450 in four conditions (T: Tumor tissue; N: Normal tissue; NoHV: no hepatitis virus infection, HBV: hepatitis B virus infection, HCV: hepatitis C virus infection; HBCV: hepatitis B and C virus infection). The eight preliminary candidate cytochromes P450 are highlighted in grey.
